# Whole-body MRI within a surveillance program for carriers with clinically actionable germline *TP53* variants - the Swedish constitutional *TP53* study SWEP53

**DOI:** 10.1186/s13053-020-0133-5

**Published:** 2020-01-13

**Authors:** Meis Omran, Lennart Blomqvist, Yvonne Brandberg, Niklas Pal, Per Kogner, Anne Kinhult Ståhlbom, Emma Tham, Svetlana Bajalica-Lagercrantz

**Affiliations:** 1 Department of Oncology-Pathology, Karolinska Institutet SE-171 77 Stockholm, Sweden AND Cancer Theme, Karolinska University Hospital, Solna, SE-171 76 Stockholm, Sweden; 2 Department of Molecular Medicine and Surgery, Karolinska Institutet, SE-171 77 Stockholm, Sweden; 3 Department of Imaging and Physiology Karolinska University Hospital, Solna, SE-171 76 Stockholm, Sweden; 4 Department of Oncology-Pathology, Karolinska Institutet, SE-171 64 Stockholm, Sweden; 5 Department of Women’s and Children’s Health, Karolinska Institutet, SE-171 77 Stockholm, Sweden; 6 Children and Women’s Health Theme, Karolinska University Hospital Solna, SE-171 76 Stockholm, Sweden; 7 Cancer Theme, Karolinska University Hospital, Solna, SE-171 76 Stockholm, Sweden; 8 Department of Molecular Medicine and Surgery, Karolinska Institutet, SE-171 77 Stockholm, Sweden; 9 Department of Clinical Genetics, Karolinska University Hospital Solna, SE-171 76 Stockholm, Sweden

**Keywords:** LiFraumeni syndrome, Germline *TP53* mutation, Hereditary breast cancer, Pathological variant, Surveillance program

## Abstract

**Background:**

The current guidelines in Sweden regarding individuals with a clinically actionable (i.e. pathogenic or likely pathogenic) germline *TP53* variant recommend patients to take part of the national Swedish P53 Study (SWEP53).

**Methods:**

The study comprises a patient registry (mandatory for all participants) and three optional parts: a biobank, a surveillance program and a psychosocial evaluation of the surveillance. All known adult eligible carriers regardless of age are offered to take part of the surveillance program offering MRI yearly of the whole-body, breast, and brain as well as breast ultrasound. A special surveillance program is offered for individuals 15–18 years old with a 50% risk of being a mutation carrier or with a verified *TP53* variation, includes ultrasound of the abdomen and urine corticosteroid profiles. Clinically motivated further examinations are performed upon need. The national inclusion is performed through the six clinical genetic units in Sweden at Umeå, Uppsala, Stockholm, Gothenburg, Linköping and Lund, and the surveillance is mainly performed through the oncology clinics.

**Results:**

To date, a total of 41 adults and 11 children have been included in the study.

**Conclusions:**

The SWEP53 is the first structured national surveillance program including radiological and clinical routines for *TP53* mutation carriers in the Scandinavian setting. The aim of this publication is to present and describe the ongoing Swedish surveillance study to encourage the initiation of similar studies and to contribute to the knowledge of adequate clinical handling of these cancer prone families.

**Trial registration:**

Trial registration number: ISRCTN13103571, retrospectively registered on 14/10/2019.

## Background

The Li-Fraumeni syndrome (LFS) was first described by Li and Fraumeni in 1969 [1]. LFS is an inherited autosomal dominant cancer syndrome caused by germline variants in the *TP53* gene. The syndrome is characterized by high risk of developing diverse tumours, mainly sarcoma, breast, brain and adrenocortical tumours [2]. Even though LFS caused by a pathogenic germline *TP53* variant is rare, pathogenic somatic *TP53* variants are the most abundant alteration identified in sporadic cancers, and is reported in 50% of all human tumours [3].

The prevalence of germline pathogenic *TP53* variants is estimated to 1:5000–1:20000 individuals [4], resulting in an estimation of between 500 and 2000 mutation carriers in the Swedish population of almost 10 million people. The actual numbers are today unknown and there is no structured *TP53* mutation screening or national registry. The estimated life-time cancer risk for female carriers of pathogenic *TP53* variants is close to 100% and the corresponding figure for males is 73% [5]. The gender difference is usually attributed to the fact that women develop breast cancer [5] [6], while others claim that female mutation carriers are more prone to cancer development regardless of the increased breast cancer risk [7]. In addition, carriers of pathogenic *TP53* variants are at risk of developing cancer at considerably younger ages (median age 25 years) than non-carriers. Up to 50% of all carriers develop a tumour before the age of 30 years [8, 9]. They are also more prone to develop multiple primary cancers [10]. Fifteen percent of all children will develop cancer before the age of 15 years [11]. In recent years, pathogenic germline *TP53* variants have also been found to cause hereditary breast cancer without childhood cancers or classic LFS [12]. The reasons behind these differences in phenotypes are today unknown, and there are no obvious differences in the genetic variants found between these two groups of patients. The European Reference Network, responsible for developing guidelines for germline *TP53* variant carriers, are therefore referring to these as hereditable *TP53* related cancer syndrome [13].

### Surveillance programs

In 2016, Villani et al [14] presented an 11-year follow-up of the first published surveillance program including whole-body MRI (WB-MRI), showing a five-year overall survival of 88.8% versus 59.6% in favour of taking part of the surveillance program compared to non-participants. These results seemed compelling. The study was, however, not randomised due to ethical aspects and there were crossovers between the groups. Further clinical studies are therefore needed to corroborate a potential increase in survival. In addition, more data is needed on the rate of benign and malignant findings in a surveillance program using MRI, as well as the feasibility of the program, including the need of further work-ups and the psychosocial impact of taking part of such a surveillance. A meta-analysis described a risk of false-positive results (defined as benign neoplasms, recurrences of pre-existing cancers, and newly diagnosed metastatic cancers) to be 42.5%, but the same study found the rate of new localized malignant findings to be 7%, of whom all were treated with a curative intention [15]. Published data from European surveillance programs [16–18] have been differently designed with regards to follow-up time and inclusion criteria regarding mutational status of individuals < 18 years old. None of the previously published data from the European studies has had a focus on the evaluation of the psychosocial impact of taking part of a surveillance program.

### Current Swedish guidelines

In Sweden, all patients with pathogenic germline *TP53* variants are offered genetic counselling and, in most regions, also the possibility of preimplantation genetic diagnosis (PGD). Besides yearly clinical checkups for all patients, female carriers are offered breast surveillance and/or prophylactic mastectomy. Historically, children in Sweden have not been offered presymptomatic genetic testing as there has not been a specific surveillance program to offer, however children at 50% risk are offered annual clinical check-ups at a pediatric clinic. The current national guidelines in Sweden now recommend that if surveillance with MRI or ultrasound is offered to*TP53* carriers, it should be performed as part of a national Swedish *TP53* study named SWEP5 in order to enable a structured evaluation. The study protocol was launched in 2017. The aim of this paper is to present the Swedish surveillance program within the SWEP53 study.

## Methods

### SWEP53 study outline

**Fig. 1:**
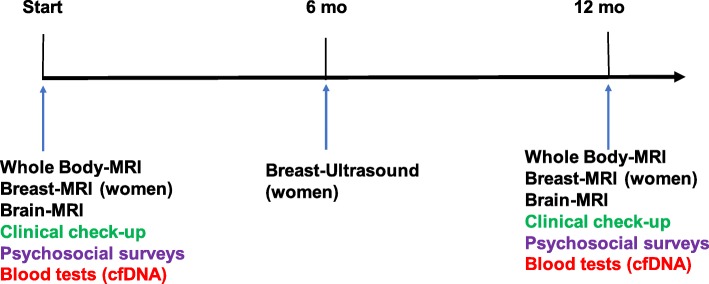
Surveillance program for individuals >15-18 years old

**Fig. 2:**
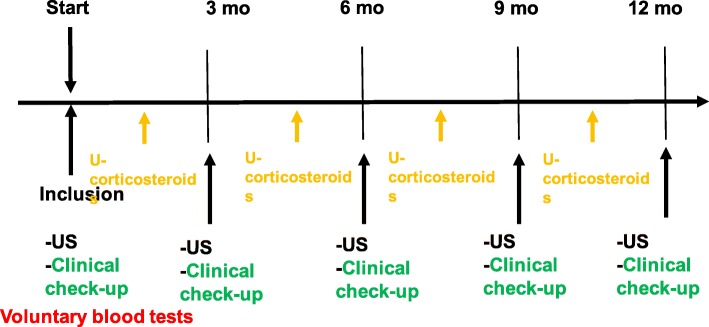
Surveillance program for individuals <18 years old

The National Swedish *TP53* Study SWEP53 consists of four parts. The first part is mandatory for inclusion in the study, whereas participation in parts 2 to 4 is voluntary:

1. National registry: All individuals who consent to participate in the study are included in a national registry containing genetic data, cancer- and family history. This registry will serve as a base for future studies.
2. A surveillance program for adults including an annual general physical examination according to a standardized protocol and whole-body MRI. Women who have not performed prophylactic mastectomy undergo yearly breast-MRI and breast ultrasound (with 6 months’ shift). Individuals up to 15 years old will be offered a protocol including ultrasound of the abdomen, urine corticosteroid profile and regular visits to a paediatrician every 3 months (Fig. 2). Those aged 15–18 years may take part in the MRI monitoring (if they are verified *TP53* mutation carriers) or continue within the childhood protocol. See Fig. 1 and 2.
3. A biobank is being established at the Karolinska University Laboratory where DNA and plasma from peripheral blood is stored. If a participant develops cancer, fibroblasts from cultured skin biopsies and DNA from tumour samples are collected. If possible, circulating tumour cells are stored. Cell free DNA from plasma will be analysed as a complement to screening with MRI, with tumour DNA as a reference. The peripheral blood DNA will be used to identify modifying factors that might explain the difference in phenotype between different families. If functional analyses are needed, RNA or protein from cultured fibroblasts will be used.
4. Patient reported outcomes (PROM) will be assessed concerning health related quality of life (HRQoL) using the Short Form (36) Health Survey [19], the Cancer worry scale [20] and the Risks and benefits of surveillance [21] in order to evaluate the psychosocial impact of participation in the surveillance program. A questionnaire of risk factors for cancer will be used to identify possible environmental modifying factors. Individuals below 18 years of age are not included in the PROM evaluation.

### Patient recruitment in the surveillance program

All individuals, regardless of age, who have been found to be carriers of clinically actionable germline *TP53* variants are offered inclusion in SWEP53 via the six regional clinical cancergenetic departments in Sweden (Umeå, Uppsala, Stockholm, Linköping, Gothenburg, and Lund). The families enrolled all have a class 5 variant, or a class 4 with a suggestive class 5 variant (due to for example segregationa analysis in the family) according to the international guidelines of the American College of Medical Genetics (ACMG). All patients have received genetic counselling prior to inclusion. The aim is to offer inclusion in SWEP53 to all known germline *TP53* families in Sweden.

Three patients were included during 2016 in order to evaluate the feasibility of the protocol.

### Designation of *TP53* variants

There are ongoing discussions if class 4 (likely pathogenic) and 5 (pathogenic) germline variants should be referred to as disease causing or clinically actionable. In the upcoming guideline for *TP53* written by ERN GENTURIS, the term disease causing is used (oral communication). However, due to the aspect that the function of risk genes is also dependent on other factors within their cellular pathways, the ability to cause a disease is highly dependent on the genetic environment for their action. Therefore, we chose to use the term clinically actionable rather than disease causing. A clinically actionable variant thus have an impact on recommendatons concerning surveillance as well as treatment.

### Inclusion criteria

- Adults > 18 years old with a verified known clinically actionable germline variant in *TP53*.
- Youths aged 15–18 years old may choose to be included in either the adult or child protocol for surveillance.
- Children 0–15 years old with a 50% risk of being a carrier (i.e. having one parent with a known clinically actionable germline *TP53* variant). In order to avoid families feeling forced to genetically test their children in order to take part in SWEP53, the Childhood Solid Tumour Working Group opted to include all children with 50% risk. Thus, the participants of the childhood protocool do not have to be confirmed carriers.

### Exclusion criteria for the surveillance part

- Contraindications to MRI.
- Co-morbidity that precludes treatment of a cancer found in the surveillance program.

This multi-centre study has been approved by the Regional Ethical Committee/Board in Stockholm, Dnr: 2015/1600–3 and amendments 2017/1527–32 and 2018/1690–32. Written informed consent is obtained from participants and/or their families.

### Study outcomes/aims

The aims of the prospective observational nationwide multi-centre SWEP53 study are to:

- Evaluate the rate of benign findings and further work-up generated by baseline whole-body MRI.
- Describe the number and pTNM stage of detected malignant lesions and the rate of curative versus palliative intention of treatment.
- pTNM staging will be described and compared to population data.
- Evaluate the psychosocial impact of taking part of the surveillance program and as well as the benefits and risks of participation over time.
- To identify genetic and environmental modifying factors that might impact the phenotype.
- To evaluate cell free DNA and circulating tumour cells (liquid biopsies) as a complement to MRI surveillance in adults with a high risk of cancer.

A Swedish clinical study group including clinical geneticists, oncologists, paediatricians, psychologist, and radiologists was formed in 2012 to plan the outline of the SWEP53. The Swedish surveillance program is modified from the so called *Toronto protocol* [22].

### Imaging

The yearly WB-MRI examinations covered initially skull base to just below patella, based on initial clinical standard settings, but were during 2017 extended to cover skull base to feet. For males and females who have undergone prophylactic mastectomy, the WB-MRI and brain MRI are performed without contrast enhancement. For females who undergo breast MRI which requires contrast enhancement, a contrast-enhanced protocol is used, (Additional file 1). The breast ultrasounds are performed in accordance with clinical procedure.

#### Image acquisition

The WB-MRI are performed on either 1.5 or 3 Tesla (T) systems. Further details regarding the imaging protocol as set up at Karolinska University Hospital are enclosed in Additional file 1. Technical improvements in MRI during the past decade, DIXON based sequences for rapid whole-body imaging as well as whole-body diffusion weighted imaging, have been implemented into the SWEP53 protocol. Contrast enhancement is only used for the women during breast MRI. The complete imaging protocol takes 90 min for women including breast MRI and 45 min for men.

#### Evaluation

The whole-body, breast and brain scans are read by two radiologists. Findings requiring further work-up are discussed at a multidisciplinary team meeting, set up by the investigators. All body sites are systematically evaluated for benign and possible malignant findings (see protocol as Additional file 2).

## Results

### Patients

So far, (December 2019), all 41 adult patients (100%) who have been informed about the study have accepted inclusion in SWEP53. All the adults, except one (98%) have chosen to participate in all four parts of the study. Eleven children have been included, of whom five are tested, and participate in the surveillance. Six are not tested, of whom three are under surveillance.

## Discussion

### The rationale behind the Swedish surveillance program

The Swedish surveillance program started to be developed during 2012 by the *Swedish Clinical TP53 Study Group* in a dialogue with national expert groups including the Swedish Society of Oncology, Swedish Society of Radiology and Swedish Childhood Solid Tumour Working Group. The Toronto protocol [22] was the only published MRI protocol for this patient group at the time (2012). A comparison between the protocols is presented in Tables 1 and 2. The Toronto protocol included colonoscopy every 2 years, whereas this is not a part of our protocol due to the lack of evidence for colorectal cancer to be a part of the germline *TP53* associated tumours. We have chosen to perform these additional examinations only if they are indicated after a review of the patient’s family history since the risk of colorectal cancer in LFS is low [14, 23–25].

**Table 1:** The Toronto and the SWEP53 protocol for children

The Toronto protocol (2011) children	Adrenocortical carcinoma	Brain tumor	Soft tissue and bone sarcoma	Leukaemia or lymphoma
	Ultrasound of abdomen and pelvis every 3–4 months	Annual brain MRI	Annual rapid total body MRI	Blood test every 4 months: complete blood count, erythrocyte sedimentation rate, lactate dehydrogenase
	Complete urinalysis every 3–4 months			
	Blood tests every 4 months: β-human chorionic gonadotropin, alpha-fetoprotein, 17-OH-progesterone, testosterone, dehydroepiandrosterone sulfate, and rostenedione			
The SWEP53 Protocol children	Ultrasound of abdomen and pelvis every 3–4 months Complete urinalysis every 3–4 months	None unless suspicion raised at the clinical check-up performed every 3 months	None unless suspicion raised at the clinical check-up performed every 3 months	None unless suspicion raised at the clinical check-up performed every 3 months

**Table 2:** The Toronto and the SWEP53 protocol for adults

The Toronto protocol (2011) Adults	Breast cancer	Brain tumor	Soft tissue and bone sarcoma	Leukaemia or lymphoma	Colon cancer	Melanoma
	Monthly breast self-examination starting at age 18 years	Annual brain MRI	Annual rapid total body MRI	Blood test every 4 months: complete blood count, erythrocyte sedimentation rate, lactate dehydrogenase	Colonoscopy every 2 years, beginning at age 40 years, or 10 years before the earliest known colon cancer in the family	Annual dermatological examination
	Clinical breast examination twice a year, starting at age 20–25 years, or 5–10 years before the earliest known breast cancer in the family		Ultrasound of abdomen and pelvis every 6 months			
	Annual mammography and breast MRI screening starting at age 20–25 years, or at earliest age of onset in the family					
	Consider risk-reducing bilateral mastectomy					
The SWEP53 Protocol Adults	Breast cancer	Brain tumor	Soft tissue and bone sarcoma	Leukemia or lymphoma	Colon cancer	Melanoma
	Monthly breast self-examination starting at age 18 years	Annual brain MRI	Whole-body MRI	Only if there are known cases in the family	Only if there are known cases in the family. 10 years before the earliest known colon cancer in the family	Annual dermatological examination
	Clinical breast examination once a year, starting at age 18					
	Annual breast MRI screening starting at age 18 and breast ultrasounds 6 month after the MRI					
	Consider risk-reducing bilateral mastectomy					

*SWEP53* The Swedish TP53 Study, *MRI* Magnetic resonance imaging

Germline *TP53*-carriers have a high risk of radiation-induced secondary malignancies and patients in SWEP53 are therefore not subjected to mammography in order to minimize exposure to radiation [8, 26, 27]. Instead, MRI and ultrasound are alternated, examining the breasts radiologically once every six months. Patients that have undergone a risk reducing breast surgery do not perform specific breast surveillance.

The Swedish Clinical *TP53* Study Group decided not to include blood tests as part of the screening, partly in order to keep the protocol as non-invasive as possible for children and partly because of the difficulties to, in the case of acute leukaemia, diagnose emerging disease prior to clinical symptoms. The time from first detectable blood clone to clinical symptoms can be very short (under a few weeks). It is also challenging to distinguish between age-related clonal haematopoiesis and leukaemia [28]. Therefore, it was decided to focus the surveillance of children on the four core tumours.

### Study design of SWEP53

The surveillance within the SWEP53 study is intended to proceed for five years, and due to prospective incusion the study i opened until 2026. The outline is similar to the Dutch LiFe-Guard study [16]. This is in contrast to the British SIGNIFY study, where patients performed one baseline screening only with matched controls [17]. The randomized clinical trial, the French LIFSCREEN study, includes a follow-up of 48 months [18]. In the present study, surveillance is offered to all known mutation carriers, because of the ethical problems included in motivating randomization in the setting of a known high life-time risk of cancer development. In addition, randomization on an individual basis would be difficult, as members of the same family could be assigned to surveillance or not, probably causing negative psychological effects. If favourable, the SWEP53 protocol will be considered as standard care in Sweden for individuals with germline *TP53* alterations.

### Rationale to include families with hereditary breast cancer in SWEP53

One of the aims of SWEP53 is to delineate the cancer risks and potential modifying factors in the two phenotypic groups of germline *TP53* carriers, namely the LFS phenotype and the hereditary breast cancer phenotype. A meta-analysis [12] found the prevalence of germline *TP53* mutations to be 7.7% in women with breast cancer < 30 years old without a LFS family history, and a 7% prevalence of *TP53* mutations amongst women < 31 years old with a HER2-positive breast cancer. Thus, *TP53* correlated breast cancer is more likely to be HER2 positive. In a review by Daly et al, 2017, the overall rate of *TP53* carriers was found to be between 0 and 4.4% amongst tested women with breast cancer. Half of these women did not fulfil LFS NCCN 2017 criteria [29]. It is still unknown if de novo mutations appearing in women with breast cancer are associated with a LFS phenotype since the family history is not informative, challenging the genetic counselling in these families. In families with pathogenic *TP53* variants seemingly associated with hereditary breast cancer, a LFS phenotype could potentially occur in the next generation. Breast cancer in young adult women is the most common core cancer in LFS. It may thus be the only LFS associated tumour appearing in generations, especially in small families with predominantly females. We therefore decided to offer similar surveillance to both groups in order to detect other tumours at an early stage.

### Finding the right level of further work-ups within SWEP53

A previous meta-analysis describes a rate of 30% of patients with actionable findings at baseline WB-MRI. The rate of benign findings, after further investigations, were 79%. The remaining were thus malignant of which 87% were individuals with new cancers [15]. Preliminary results indicate a similar rate in SWEP53. To manage these, a consistent follow-up care flow is important. There is a need to further evaluate the rate of total work-up and secondary findings. The high-risk for cancer development in this group has implications for the need for further work-up (i.e. we might be more willing to take an extra cytology or biopsy sample compared to a normal patient). A finding of unclear significance should perhaps be considered a cancer until proven to be benign. In addition, *TP53* mutation carriers are more prone to develop treatment induced malignancies with implications regarding choice of therapy [26, 27, 30]. Special considerations are also needed in order to avoid unnecessary exposure to irradiation. It is therefore crucial to establish a local multidisciplinary on-site team including oncologists, radiologists, paediatricians, genetic counsellors and clinical geneticists in order to offer personalized care.

### Inventory of known *TP53* carriers in Sweden

There is no national registry containing information on all known *TP53* carriers and thus the incidence in Sweden is unknown. Only 25 adult carriers are known in the Stockholm area, much less than the expected 100–400 based on the supposed incidence 1:5000–1:20000 [4], suggesting a huge under-diagnosis of this condition. This is slowly changing as molecular diagnosis including tumour sequencing as part of a pre-treament predictive screening with panel testing is being introduced in clinical routine analysis. Moreover, in Sweden, women with early-onset breast cancer are now routinely screened for germline alterations with a panel including *TP53*, thus identifying previously unknown carriers in families with hereditary breast cancer rather than the classical LFS tumour panorama.

### Genetic counselling and psychological aspects

The patients who are offered participation in the SWEP53 study have undergone genetic counselling at the departments of clinical genetics at the participating sites prior to testing and when the results was presented. These patients have experiences of cancer in close relatives, often with fatal outcomes. A relatively low prevalence of clinically relevant levels of distress have been reported in patients with LFS after receiving the results of genetic testing [20]. About 25% of the patients were, however, in need of professional psychosocial support, but this was irrespective of their carrier status and previous history of cancer. Regular breast cancer surveillance has not been found to be associated with psychological burden [21]. A study examining the psychosocial benefits of a comprehensive whole-body screening program for patients with *TP53* mutations suggested that the program provided psychological benefit independent of the impact on cancer morbidity and mortality associated with the syndrome [31]. In the SWEP53 study, the patients’ evaluation of the program is important to ascertain if the surveillance program adds worry and emotional problems among the participants. We are studying the possible psychosocial consequences of participation, and also perceived benefits and barriers to participation in the program. The participants are offered psychological support if needed during the study period. The results will form the basis of psychosocial care for patients with hereditary *TP53* variants undergoing surveillance.

### Children

In several published surveillance programs WB-MRI screening has been included for children, even though it requires general anaesthesia in young ages [18, 32, 33]. In these programs, genetic testing of *TP53* in participating children is required. In the SWEP53 study, included children need to have a parent with a pathogenic *TP53* variant, but genetic testing is not required. This inclusion criteria was discussed at length with the Childhood Solid Tumour Working Group and the local paediatricians involved in the heath care of children with Li-Fraumeni syndrome. Since there is not yet robust evidence for the benefits of surveillance in children with *TP53* variants, we felt it was unethical to demand genetic testing for inclusion in a research study. Presymptomatic testing of healthy children has not been routinely offered to children in Sweden, although this is slowly changing due to increased awareness of genetic testing among health care professionals and the general public. Knowing the mutational status without being able to affect it may have serious implications on family dynamics and psychological development in the child and should therefore not be part of a research study. If a surveillance program shows certain clinical benefit and is introduced in clinical routine for children, then genetic testing will be performed in order to identify children who have inherited a *TP53* variant and are thus at risk for cancer.

The children are not asked to complete questionnaires. To the best of our knowledge, validated psychosocial questionnaires for children are not available. In addition, there is a risk that asking children about possible negative psychological consequences might lead to a focus on their possible risk for cancer.

## Conclusions

The SWEP53 study is ongoing and open for inclusion for all patients in Sweden with a clinically actionable germline variant in *TP53,* regardless of the phenotypic differences amongst the families. Children at a 50% risk of being a mutation carrier are also eligible for inclusion. These families are thereby offered to take part in the first nationwide surveillance study that also includes patient related outcomes. By establishing a registry and by collecting DNA, plasma and tumour samples, and fibroblasts, we are also creating opportunities for further studies aimed at understanding the mechanisms behind the phenotype variation within the hereditary *TP53* cancer syndrome and how to best tailor follow-up.

When it comes to hereditary *TP53* variants, each country have a limited number of carriers. Therefore it would be valuable to have cross border studies. However, due to the complexity of the surveillance and the variety of clinical guidelines among countries, we developed a national study. With this publication, we hope to facilitate the establishment of such programs harmonizing with SWEP53 to extend the cohort and thereby increasing the knowledge of the clinical handling of cancer prone carriers, and improving the survival and quality of life.

## Supplementary information

**Additional file 1.** Imaging protocol, whole-body MRI.

**Additional file 2.** Standardised protocol for evaluation of whole-body MRI within SWEP53.

## Data Availability

not applicable (no data).

## Abbreviations

cfDNA: Cell free plasma DNA
LFS: The li fraumeni syndrome
PROM: Patient reported outcomes
SWEP53: The swedish *TP53* study
*TP53*: Tumour protein 53
WB-MRI: Whole-body magnetic resonance imaging
