# Supplementary material for: Whole-body MRI within a surveillance program for carriers with clinically actionable germline TP53 variants - the Swedish constitutional TP53 study SWEP53
Source: Hered Cancer Clin Pract. 2020 Jan 13;18:1. doi: 10.1186/s13053-020-0133-5 (PMC6958585; doi:10.1186/s13053-020-0133-5)
Supplement: Supplementary file 1 — Additional file 1. Imaging protocol, whole-body MRI. [file 13053_2020_133_MOESM1_ESM.pdf]

Supplement 1.

Imaging protocol, WB-MRI.

**WB-MRI**	**1.5 T (Siemens Area)**				
Sequence	SSFSE/HASTE Fat ST		DIXON		T2/EPI b50_400_800
	T2		T1		DWI
Orientation	Axial		Axial		Axial
TR/TE (ms)	1000/82		165/		5600/60
Field of view (mm)	400x275		416/315		420/338
Matris	256/123		256/134		192/156
Slice thickness/fsk (mm)	5/1		5/1		5/1
NEX	1		1		4
Number of slice partitions	208		216		210
Pixel bandwidth	500		475		1735
Coverage	Neck-below knees		Neck-below knees		Neck-below knees
Scan time					

Imaging protocol details

The imaging protocol consist of DIXON T1-weighted, single-shot TIRM (turbo inversion recovery magnitude), and DWI-EPI (diffusion weighted echo planar imaging) with three different b-values (50, 400 and 800), all in a transversal plane. For the brain, a transversal T1-weighted MPRAGE (Magnetization Prepared Rapid Gradient Echo) is used. For the women, we add a breast protocol before and after intravenous contrast agent (Gadolinium), which is also used for the brain MRI. Male patients do not receive any contrast agent.
