# Supplementary material for: Whole-body MRI within a surveillance program for carriers with clinically actionable germline TP53 variants - the Swedish constitutional TP53 study SWEP53
Source: Hered Cancer Clin Pract. 2020 Jan 13;18:1. doi: 10.1186/s13053-020-0133-5 (PMC6958585; doi:10.1186/s13053-020-0133-5)
Supplement: Supplementary file 2 — Additional file 2. Standardised protocol for evaluation of whole-body MRI within SWEP53. [file 13053_2020_133_MOESM2_ESM.pdf]

Ver 1 June 25 2018 LB

Standardised protocol for evaluation of whole body MRI within SWEP53

Hospital

Date of imaging (year-mm-dd)

Radiologist

	Not examined	Normal y	Benign finding - no further work-up, describe	Size (mm)	Unclear finding – further work-up required, describe	Size (mm)	Suspect malignant finding – further work-up required	Size (mm)	Comment
Breast									
Brain									
Thyroid									
Cervical									
Thorax									
Mediastinum									
Liver									
Bile ducts									
Pancreas									
Spleen									
Adrenal glands									
Kidneys									
Retroperitoneum									
Urine bladder									
Uterus/prostate									
Vagina/Vesicles									
External genitalia									
Soft tissue									
Cervical									
Thorax									
Upper abdomen									
Lower abdomen									
Upper extremities									
Lower extremities									
Skeletal									
Cervical									
Thorax									
Upper abdomen									
Lower abdomen									
